# Supplementary material for: Sources of stress, coping strategies and associated factors among Vietnamese first-year medical students
Source: PLoS One. 2024 Jul 31;19(7):e0308239. doi: 10.1371/journal.pone.0308239 (PMC11290621; doi:10.1371/journal.pone.0308239)
Supplement: S1 Checklist — (DOCX) [file pone.0308239.s001.docx]

STROBE Statement—checklist of items that should be included in reports of observational studies

|  | Item No. | Recommendation | Page  No. | Relevant text from manuscript |
| --- | --- | --- | --- | --- |
| **Title and abstract** | 1 | (*a*) Indicate the study’s design with a commonly used term in the title or the abstract | 02 | We conducted a cross-sectional study |
|  |  | (*b*) Provide in the abstract an informative and balanced summary of what was done and what was found | 02 - 03 | We conducted a cross-sectional study with 409 first-year students at the University of Medicine and Pharmacy, Vietnam. The survey comprised questions of socioeconomic status, stress-related issues, the six sources of stress (using Higher Education Stress Inventory), and the nine coping strategies (using Brief Coping Orientation to Problems Experienced). Factor analysis of HESI scores identified six sources of stress. Two-thirds of first-year medical students reported moderate to high levels of stress. “Worries about future competence/endurance” was the most concerned stressor, followed by “Academic workload”, and “Financial concerns”. The participants reported high frequency of utilization “Self-distraction”, “Problem-solving” and “Social support” when confronting stress. |
| Introduction | | | |  |
| Background/rationale | 2 | Explain the scientific background and rationale for the investigation being reported | 03 - 04 | Being exposed to prolonged and high levels of stress may lead to negative consequences: cognitive and emotional burden, psychological distress, dropping out of school, low quality of life and decreased empathy with patients, which all may result in medical errors and may influence the quality of health care service in the wide aspect. Research has suggested that medical students experience stress from different sources, which could be from academic factors, psychosocial factors with emphasis on financial issues, teacher-student relationships, and high expectations from significant others and self-. Early identification of these stressors could help tailor appropriate prevention and intervention programs to tackle the problematic psychological issues among this high-risk population.  There’re different ways to categorize coping strategies, consisting of problem-focused or emotion-focused, adaptive or maladaptive, approaching or avoiding. Adopting appropriate strategies would help students cope better and healthier with stressors, minimizing the negative consequences from stressors. Hence, it is essential to first understand what sources of stress that medical students are facing, then how they are coping with a variety of stressors. |
| Objectives | 3 | State specific objectives, including any prespecified hypotheses | 04 | Our aims are to examine the sources of stress among first-year medical students, the frequencies of their coping strategies, and to explore the relationships between other factors with specific sources of stress and coping strategies employed by these students. |
| Methods | | | |  |
| Study design | 4 | Present key elements of study design early in the paper | 05 | We conducted a cross-sectional study.  In this study, we invited all first-year students.  Participants filled in a self-administered survey two months after school admission.  The survey included: (i) Demographic information (Age, Sex, Parental education status, Part-time job); (ii) Self-rated stress level, acute stress event, physical issues, psychological issues, Covid-19-related stress; (iii) Higher Education Stress Inventory (HESI) to assess the sources of stress; (iv) Brief Coping Orientation to Problems Experienced (Brief COPE) to assess the coping strategies. |
| Setting | 5 | Describe the setting, locations, and relevant dates, including periods of recruitment, exposure, follow-up, and data collection | 05 | We conducted a cross-sectional study among the undergraduate students at Faculty of Medicine, University of Medicine and Pharmacy, Ho Chi Minh City, Vietnam.  Participants filled in a self-administered survey two months after school admission.  Data were collected from 12th November 2020 to 22nd December 2020, and have been accessed for data analysis since 24th December 2020. |
| Participants | 6 | (*a*) *Cohort study*—Give the eligibility criteria, and the sources and methods of selection of participants. Describe methods of follow-up  *Case-control study*—Give the eligibility criteria, and the sources and methods of case ascertainment and control selection. Give the rationale for the choice of cases and controls  *Cross-sectional study*—Give the eligibility criteria, and the sources and methods of selection of participants | 05 | In this study, we invited all first-year students. Participants provided written informed consents before participating in the study. Students with informed consent and completed data were included in the study with no explicit exclusion criteria were employed. |
|  |  | (*b*) *Cohort study*—For matched studies, give matching criteria and number of exposed and unexposed  *Case-control study*—For matched studies, give matching criteria and the number of controls per case |  |  |
| Variables | 7 | Clearly define all outcomes, exposures, predictors, potential confounders, and effect modifiers. Give diagnostic criteria, if applicable | 07, 09, 10 | The score of each source of stress was the average score of their related items, ranging from 1.0 to 4.0.  The score of each coping strategy was the average score of their related items, ranging from 1.0 to 4.0.  Level of stress was scored from 1 to 3 for low to high level, respectively. Mother and father educational levels were categorized into three levels. Part-time job, acute stress event, physical stress, psychological stress, and Covid-19-related stress were binary variables with responses of yes or no. |
| Data sources/ measurement | 8* | For each variable of interest, give sources of data and details of methods of assessment (measurement). Describe comparability of assessment methods if there is more than one group | 06, 07, 08, 09 | The survey included: (i) Demographic information (Age, Sex, Parental education status, Part-time job); (ii) Self-rated stress level, acute stress event, physical issues, psychological issues, Covid-19-related stress; (iii) Higher Education Stress Inventory (HESI) to assess the sources of stress; (iv) Brief Coping Orientation to Problems Experienced (Brief COPE) to assess the coping strategies.  From 33 items from the original version, we performed Exploratory Factor Analysis (EFA) and Confirmatory Factor Analysis (CFA) to get the revised scale with 20 items, categorized into six sources of stress.  The six sources of stress included:  1. Mismatch in professional role expectations (from four items “The training demands that I join in situations that I find unethical”; “The professional role presented in the training conflicts with my personal view”; “I feel that I am less well treated because of my ethnic background”; and “I feel that I am less well treated because of my sex”).  2. Worries about future competence/endurance (from three items “I worry about long working hours and responsibilities in my future career”; “The insight I have had into my future profession has made me worries about the stressful workload”; “I am worried that I will not acquire all the knowledge needed for my future profession”).  3. Financial concerns (from three items “As a student, my financial situation is a worry”, “I am worried about my future economy and my ability to repay students loans”; “I am worried about accommodation”).  4. Academic workload (from three items “The literature is too difficult and extensive”; “The space of studies is too high”; “Studies control my life and I have little time for other activities”).  5. Low identity of medical profession (from three items “I am satisfied with my choice of career” – reversed score; “I am proud of my future profession” – reversed score; “I am able to influence my studies” – reversed score).  6. Non-supportive educational environment (from four items “Student union activities promote a sense of community and contribute to a better working environment for students” – reversed score; “The teachers often give feedback on students’ knowledge and skills” – reversed score; “I feel that the training is preparing me well for my future profession” – reversed score; “My fellow students support me” – reversed score)  Our findings from EFA and CFA of the original Brief COPE revealed the revised 27-item scale with 9 categories of coping strategies.  The nine coping strategies included:  1. Problem solving (from seven items “I’ve been taking action to try to make the situation better”; “I’ve been concentrating my efforts on doing something about the situation I’m in”; “I’ve been trying to come up with a strategy about what to do”; “I’ve been thinking hard about what steps to take”; “I’ve looking for something good in what is happening”; “I’ve been learning to live with it”; “I’ve been trying to see it in a different light, to make it seem more positive”).  2. Social support (from four items “I’ve been getting help and advice from other people”; “I’ve been getting emotional support from others”; “I’ve been getting comfort and understanding from someone”; “I’ve been trying to get advice or help from other people about what to do”).  3. Avoidance (from four items “I’ve been giving up the attempt to cope”; “I’ve been refusing to believe that it has happened”; “I’ve been giving up trying to deal with it”; “I’ve been saying to myself ‘this isn’t real’”).  4. Substance use (from two items “I’ve been using alcohol or other drugs to make myself feel better”; “I’ve been using alcohol or other drugs to help me get through it”).  5. Self-blame (from two items “I’ve been criticizing myself”; “I’ve been blaming myself for things that happened”).  6. Religion (from two items “I’ve been praying or meditating”; “I’ve been trying to find comfort in my religion or spiritual beliefs”)  7. Humor (from two items “I’ve been making fun of the situation”; “I’ve been making jokes about it”).  8. Venting (from two items “I’ve been saying things to let my unpleasant feeling escape”; “I’ve been expressing my negative feelings”).  9. Self-distraction (from two items “I’ve been doing something to think about it less, such as going to movies, watching TV, reading, daydreaming, sleeping or shopping”; “I’ve been turning to work or other activities to take my mind off things”).  The score of each coping strategy was the average score of their related items, ranging from 1.0 to 4.0. |
| Bias | 9 | Describe any efforts to address potential sources of bias | 05 | we invited all first-year students. Participants provided written informed consents before participating in the study; and those with informed consent were included in the study with no explicit exclusion criteria were employed.  The survey was anonymous with no information to identify the participants. Data were collected from 12th November 2020 to 22nd December 2020, and have been accessed for data analysis since 24th December 2020. |
| Study size | 10 | Explain how the study size was arrived at | 05 | In this study, we invited all first-year students.  There were 409 students completing the questionnaire, making the participation rate 97%. |

Continued on next page

| Quantitative variables | 11 | Explain how quantitative variables were handled in the analyses. If applicable, describe which groupings were chosen and why | 10 | Descriptive statistics (mean, standard deviation, and percentage) were used for calculating the frequencies and proportions of demographic variables, sources of stress, and coping strategies.  Level of stress was scored from 1 to 3 for low to high level, respectively. Mother and father educational levels were categorized into three levels. Part-time job, acute stress event, physical stress, psychological stress, and Covid-19-related stress were binary variables with responses of yes or no. |
| --- | --- | --- | --- | --- |
| Statistical methods | 12 | (*a*) Describe all statistical methods, including those used to control for confounding | 10 | To assess the factors associated with sources of stress and coping strategies, multiple linear regressions were estimated. For each source of stress, the regression model included all sociodemographic factors: sex, age, parental educational levels, part-time job, level of perceived stress, and other stress-related variables (acute stress event, physical stress, psychological stress, Covid-19-related stress). Similarly for each coping strategy, we run different regression models that included different sources of stress and all the above factors. |
|  |  | (*b*) Describe any methods used to examine subgroups and interactions |  |  |
|  |  | (*c*) Explain how missing data were addressed | 05 | Students with informed consent and completed data were included in the study.+- |
|  |  | (*d*) *Cohort study*—If applicable, explain how loss to follow-up was addressed  *Case-control study*—If applicable, explain how matching of cases and controls was addressed  *Cross-sectional study*—If applicable, describe analytical methods taking account of sampling strategy |  |  |
|  |  | (*e*) Describe any sensitivity analyses |  |  |
| Results | | | | |
| Participants | 13* | (a) Report numbers of individuals at each stage of study—eg numbers potentially eligible, examined for eligibility, confirmed eligible, included in the study, completing follow-up, and analysed | 10 | Among the 409 participants, |
|  |  | (b) Give reasons for non-participation at each stage |  |  |
|  |  | (c) Consider use of a flow diagram |  |  |
| Descriptive data | 14* | (a) Give characteristics of study participants (eg demographic, clinical, social) and information on exposures and potential confounders | 10 | the mean age was 18.1 with a standard deviation of 0.38. Approximately 40% of participants were female. Most of the participants’ parents finished their high school or higher. More than 20% of students reported experiencing either physical issues or psychological issues, while less than 10% had stress due to Covid-19. There were 231 students (57%) reported a moderate level of stress, and 36 students (8.9%) had a high level of stress. |
|  |  | (b) Indicate number of participants with missing data for each variable of interest |  |  |
|  |  | (c) *Cohort study*—Summarise follow-up time (eg, average and total amount) |  |  |
| Outcome data | 15* | *Cohort study*—Report numbers of outcome events or summary measures over time |  |  |
|  |  | *Case-control study—*Report numbers in each exposure category, or summary measures of exposure |  |  |
|  |  | *Cross-sectional study—*Report numbers of outcome events or summary measures | 12, 13 | “Worries about future competence/endurance” had the highest mean score among 409 participants (3.02±0.64), while “Mismatch in professional role expectations” had the lowest score (1.60±0.53). “Financial concerns” and “Academic workloads” were also significant sources of stress among first-year students in this study, with the mean scores of 2.65 and 1.86, respectively. The other stressors with less concerns were “Low identity of medical profession” (mean score: 1.87) and “Non-supportive educational environment” (mean score: 1.80).  Regarding coping strategies employed by the study population, Self-distraction was the most frequently reported among these 409 students (2.80 ± 0.68). However, Problem-solving and seeking Social support were predominant the other less adaptive strategies, with the scores were 2.72 (0.53) and 2.62 (0.70), respectively. Self-blame also had a high score (2.52±0.74), i.e., it appears quite frequently among the participants. Avoidance (1.87±0.55) and substance-use (1.27±0.55) were the least frequent strategies adopted to cope with stress. |
| Main results | 16 | (*a*) Give unadjusted estimates and, if applicable, confounder-adjusted estimates and their precision (eg, 95% confidence interval). Make clear which confounders were adjusted for and why they were included | 14, 15 | Table 5 illustrated a variety of associated factors with coping strategies utilized by the participants. Students with “Low identity of medical profession” were less likely to seek social support (β=-0.25). Meanwhile, students with stressors due to “Mismatch in professional role expectations” tend to have both adaptive and maladaptive strategies: Humor (β=0.25), Religion (β=0.3), Avoidance (β=0.22), and Substance use (β=0.25). Substance use was also found positively associated with stressors from “Non-supportive educational environment”, “Having physical issues” and “Having part-time jobs”. On the other hand, Self-blame was reported higher frequency among students with “Worries about future competence/endurance” (β=0.14), “Financial concerns” (β=0.17), “Academic workload” (β=0.18), “High perceived stress level” (β=0.20). Male student tent to adopt humor strategy (β=0.25, p=0.02), while less likely to utilize religious practices (β= -0.21, p=0.01). |
|  |  | (*b*) Report category boundaries when continuous variables were categorized |  |  |
|  |  | (*c*) If relevant, consider translating estimates of relative risk into absolute risk for a meaningful time period |  |  |

Continued on next page

| Other analyses | 17 | Report other analyses done—eg analyses of subgroups and interactions, and sensitivity analyses |  |  |
| --- | --- | --- | --- | --- |
| Discussion | | | | |
| Key results | 18 | Summarise key results with reference to study objectives | 16, 17, 18, 19, 20 | The findings reveal that two-thirds of the participants rated their stress levels were moderate to high level after admission two months. This is comparable to other studies from Vietnam and other medical school in other countries. The study of Quynh et al. in 2020 showed almost 50% of students have problems with stress. Another study among 686 students found that 61.4% of the medical students reported different degrees of stress. However, only 2.4% of students rated their stress level as high in that study, whereas our finding showed 8.9% of the participants had high level of stress. Also in that study, the most prevalent sources of stress were from academic performance, including Test/exam (99% students), Falling behind in reading schedule (~97% students), Getting poor marks (~93% students), or Heavy workload (85.2% students). “Feeling of incompetence” was reported from 78.4%, while poor motivation to learn met in 48% of students (21). The sources of stress reported in our studies were measured by the revised 20-items HESI scale, which showed that Worries about future incompetence/endurance was the highest ranked stressor of the participants. “Academic workload” came as the second significant source of stress, followed by “Financial concerns”. One remarkable observation was that the scores of the remaining three sources of stress were much lower than the first ranked three stressors above. This suggests that medical undergraduate students face a variety of stressors, which are not just limited to academic burden, but encompass their self-expectations and motivations to practice medical profession in the future.  Mismatch in professional role expectations with their inner conflicts to educational activities, served as other sources of stress. Pursuing medical practice requires students to understand patients’ suffering and be willing to provide necessary treatment with professionalism and empathy. Accordingly, training activities would build up professional roles with specific expectations, including but not limited to being authentic, ethical, and respectful. Without the necessary supportive system, students would feel frustrated and stressed, particularly in their first year entering medical education. In our study, “Non-supportive educational environment” includes both the school environment and teacher-student relationships. Previous studies focus on students’ experiences of homesick, difficulties in adapting new environments, as well as lacks of contact with family; or competitive, cold and impersonal attitudes. Since HESI was not designed specifically for medical education settings, we do not measure other sources of stress from medical training: facing illness or death of patients, parental wish for you to study medicine.  The role of gender when investigating stressors in medical students has long been studied, with inconsistent findings. In our study, female students and students were more likely to get stress due to their low identity of medical profession, but not other sources of stress. This finding was aligned with other studies as non-male gender has been identify as risk factor of medical students’ distress, particular stressor due to academic workload. On the other hand, study of Yogesh et al of 100 first-year medical students showed that stress levels due to academic and interpersonal issues were lower in female compared to their male counterpart. Another study of Sadiq et al revealed higher levels of all stressors among female students, but no significant correlations between gender and sources of stress was found. Furthermore, our findings showed that students experiencing acute stress event tend to be stressed due to financial concerns, while students with part-time jobs were more likely to report stress of “Low identity of medical profession”. We have not found any evidence of these social factors associated with stressors among medical students in the available research. Understanding the association of these socioeconomic factors and different sources of stress would help school administrators analyze and figure out appropriate approaches to support these students. Further studies may need to explore more these relationships.  Regarding coping strategies, Self-distraction was the most frequently used among 409 participants. However, active coping strategies (Problem-solving, seeking for Social support, Venting) were also popular in this study population. This was aligned with findings of other studies on coping strategies of medical students. On the other hand, Humor and Religion (Religious practice) were reported with lower frequency. Religious practicing was one of the most adopted coping strategies in some countries, but not in our study population. Positive religious practice has been proved positively effect on students’ resilience and their mental health. Among the maladaptive coping strategies, Self-blame had a relatively high score, whereas Substance-use and Avoidance were found not frequently adopted by the participants. These are positive findings among the first-year students since previous studies conducted in the United Kingdom and in Nepal found “Substance use” as a very common coping strategies among medical students. However, under-reporting cannot be ruled out. Since substance-use was regarded as moral issue in Vietnam society, students might not be open about it despite the anonymity and confidentiality nature of our surveys.  Associations between different factors and coping strategies were observed. Overall, the strength of associations was low to moderate, yet in the expected directions. This study found that the levels of perceived stress was only linked to Self-blame, which was discrepant with the previous findings that high level of stress would increase the use of maladaptive coping strategies besides self-blame, including substance use, denial, wishful thinking, behavioral disengagement (35, 36). Male students were more likely to adopt humor strategy than their female counterpart, while less likely to have religious practice. This was similar to the results of a study conducted in a sample of 94 medical students in their third year about the use of humor strategy(37). This also added new insights into the previous studies about coping strategies’ differences among male and female medical students. Though the common findings were that female students tent to adopt emotional and instrumental support, venting and self-distraction, we did not find the significant associations between students’ gender with these coping strategies.  Furthermore, we found that specific sources of stressors were linked to specific strategies when students confront stress. Either “Worries about future competence/endurance”, “Financial concerns” or “Academic workload” was found positively associated with Self-blame. On the other hand, when students had “Low identity of medical profession”, they were less likely to seek social support (emotional or instrumental support). While “Mismatch in professional role expectations” was positively associated with the frequency of adoption Humor, Religion (Religious practice), Avoidance, and Substance use strategies; “Non-supportive educational environment” only significantly associated with Substance use among the participants. Finally, students with physical issues were more likely to use substances to deal with stress compared to those reported no stress due to physical issues. Provided that these associations between stressors and coping strategies have not been studied in the field of medical training, our findings were novel and would shed the light on approaches to enhance students’ coping strategies to deal with different sources of stress. |
| Limitations | 19 | Discuss limitations of the study, taking into account sources of potential bias or imprecision. Discuss both direction and magnitude of any potential bias | 20 | The study has some limitations, including the nature of cross-sectional study could not draw any causal relationship between the sources of stressors and coping strategies adopted by the participants. Also, when measuring the frequency of coping strategies, the questions did not specify the sources of stress. Hence, we could not imply the high frequency of any coping strategy due to any specific source of stressors. However, the strength of this study was the participation of 97% of first-year students in a school, enhancing the representative of the study population. Furthermore, the instruments used were validated in this study population, ensuring the validity and reliability of the scales. |
| Interpretation | 20 | Give a cautious overall interpretation of results considering objectives, limitations, multiplicity of analyses, results from similar studies, and other relevant evidence | 20, 21 | Medical students were exposed to a variety of stressors since their first year of training, which resulted in two-thirds of students reporting moderate to high level of stress in this study. “Worries about future competence/endurance” was the most concerned stressor, followed by “Academic workload”, and “Financial concerns”. The participants reported high frequency of utilization “Self-distraction”, “Problem-solving” and seeking “Social support” when dealing with stress. The findings revealed significant associated factors of sources of stress and coping strategies adopted by first-year medical students. |
| Generalisability | 21 | Discuss the generalisability (external validity) of the study results |  |  |
| Other information | |  | | |
| Funding | 22 | Give the source of funding and the role of the funders for the present study and, if applicable, for the original study on which the present article is based |  |  |

*Give information separately for cases and controls in case-control studies and, if applicable, for exposed and unexposed groups in cohort and cross-sectional studies.

**Note:** An Explanation and Elaboration article discusses each checklist item and gives methodological background and published examples of transparent reporting. The STROBE checklist is best used in conjunction with this article (freely available on the Web sites of PLoS Medicine at http://www.plosmedicine.org/, Annals of Internal Medicine at http://www.annals.org/, and Epidemiology at http://www.epidem.com/). Information on the STROBE Initiative is available at www.strobe-statement.org.
