# Supplementary material for: Sources of stress, coping strategies and associated factors among Vietnamese first-year medical students
Source: PLoS One. 2024 Jul 31;19(7):e0308239. doi: 10.1371/journal.pone.0308239 (PMC11290621; doi:10.1371/journal.pone.0308239)
Supplement: Attachment — Submitted filename: PONE-D-23-15195_reviewed.pdf [file pone.0308239.s002.pdf]

PLOS ONE

Sources of stress, coping strategies and associated factors among Vietnamese first-year medical students

--Manuscript Draft--

**Manuscript Number:**	PONE-D-23-15195
**Article Type:**	Research Article
**Full Title:**	Sources of stress, coping strategies and associated factors among Vietnamese first-year medical students
**Short Title:**	Stress and coping strategies in Vietnamese medical students
**Corresponding Author:**	Song-Lih Huang, MD., PhD.<br>National Yang Ming Chiao Tung University<br>Taiwan TAIWAN
**Keywords:**	Sources of stress; Coping strategies; Medical students; Higher Education Stress Inventory, Brief COPE
**Abstract:**	Objectives: This study aims to examine the sources of stress among first-year medical students; the frequency of their coping strategies; the factors associated with specific stressors and specific coping strategies adopted by the participants.<br>Methods: We conducted a cross-sectional study with 409 first-year students at the University of Medicine and Pharmacy, Vietnam. The survey comprised questions of socioeconomic status, stress-related issues, the six sources of stress (using Higher Education Stress Inventory), and the nine coping strategies (using Brief Coping Orientation to Problems Experienced). Factor analysis of HESI scores identified six sources of stress.<br>Results: Among the six sources of stress, "Worries about future competence/endurance" had the highest mean score (3.02±0.64), while "Mismatch in professional role expectations" had the lowest score (1.60±0.53). "Financial concerns" and "Academic workloads" were also significant sources of stress. Regarding coping strategies, Self-distraction was most frequently adopted by the participants (2.80 ± 0.68). Problem-solving (2.72±0.53) and seeking Social support (2.62±0.70) were also common adaptive strategies. Avoidance (1.87±0.55) and substance-use (1.27±0.55) were the least frequent strategies. Students who experienced acute stress event were more likely to have financial concerns compared to others. Substance use was positively associated with stressors from "Non-supportive educational environment", "Having physical issues" and "Having part-time jobs". Self-blame was more frequent among students with "Worries about future competence/endurance", "Financial concerns", "Academic workload", and "High perceived stress level". Male student tended to adopt humor strategy (β=0.25), while less likely to utilize religious practices (β= -0.21, p=0.01).<br>Conclusions: Two-thirds of first-year medical students reported moderate to high levels of stress. "Worries about future competence/endurance" was the most concerned stressor, followed by "Academic workload", and "Financial concerns". The participants reported high frequency of utilization "Self-distraction", "Problem-solving" and "Social support" when confronting stress. The findings may help inform the school management to better support students' well-being.
**Order of Authors:**	Tan Nguyen, M.D., MSc.
	Song-Lih Huang, MD., PhD.
	Christy Pu, PhD
	Alexander Waits, PhD
	Tuan D Tran, MD., PhD.
	Yatan Pal Singh Balhara, PhD
	Quynh Thi Vu Huynh, MD., PhD.
**Additional Information:**	

Powered by Editorial Manager® and ProduXion Manager® from Aries Systems Corporation

Question	Response
**Financial Disclosure**<br><br>Enter a financial disclosure statement that describes the sources of funding for the work included in this submission. Review the submission guidelines for detailed requirements. View published research articles from *PLOS ONE* for specific examples.<br><br>This statement is required for submission and **will appear in the published article** if the submission is accepted. Please make sure it is accurate.<br><br>**Unfunded studies**<br>Enter: *The author(s) received no specific funding for this work.*<br><br>**Funded studies**<br>Enter a statement with the following details:<br>• Initials of the authors who received each award<br>• Grant numbers awarded to each author<br>• The full name of each funder<br>• URL of each funder website<br>• Did the sponsors or funders play any role in the study design, data collection and analysis, decision to publish, or preparation of the manuscript?<br>• **NO** - Include this sentence at the end of your statement: *The funders had no role in study design, data collection and analysis, decision to publish, or preparation of the manuscript.*<br>• **YES** - Specify the role(s) played.<br><br>* typeset	The author(s) received no specific funding for this work.
**Competing Interests**<br><br>Use the instructions below to enter a competing interest statement for this submission. On behalf of all authors, disclose any competing interests that could be perceived to bias this work—acknowledging all financial support and any other relevant financial or non-financial competing interests.	The authors have declared that no competing interests exist.

Powered by Editorial Manager® and ProduXion Manager® from Aries Systems Corporation

This statement is required for submission and **will appear in the published article** if the submission is accepted. Please make sure it is accurate and that any funding sources listed in your Funding Information later in the submission form are also declared in your Financial Disclosure statement.<br><br>View published research articles from *PLOS ONE* for specific examples.<br><br>**NO authors have competing interests**<br><br>Enter: *The authors have declared that no competing interests exist.*<br><br>**Authors with competing interests**<br><br>Enter competing interest details beginning with this statement:<br><br>*I have read the journal's policy and the authors of this manuscript have the following competing interests: [insert competing interests here]*<br><br>* typeset	
**Ethics Statement**<br><br>Enter an ethics statement for this submission. This statement is required if the study involved:<br><br>• Human participants<br>• Human specimens or tissue<br>• Vertebrate animals or cephalopods<br>• Vertebrate embryos or tissues<br>• Field research<br><br>Write "N/A" if the submission does not require an ethics statement.<br><br>General guidance is provided below. Consult the submission guidelines for detailed instructions. **Make sure that all information entered here is included in the Methods section of the manuscript.**	This study was approved by the IRB of National Yang Ming University (Number: YM109074E) and University of Medicine and Pharmacy (Number: 377/UMP-BOARD. All participants provided written informed consents before participating in this study.

Powered by Editorial Manager® and ProduXion Manager® from Aries Systems Corporation

**Format for specific study types**<br><br>**Human Subject Research (involving human participants and/or tissue)**<br>• Give the name of the institutional review board or ethics committee that approved the study<br>• Include the approval number and/or a statement indicating approval of this research<br>• Indicate the form of consent obtained (written/oral) or the reason that consent was not obtained (e.g. the data were analyzed anonymously)<br><br>**Animal Research (involving vertebrate animals, embryos or tissues)**<br>• Provide the name of the Institutional Animal Care and Use Committee (IACUC) or other relevant ethics board that reviewed the study protocol, and indicate whether they approved this research or granted a formal waiver of ethical approval<br>• Include an approval number if one was obtained<br>• If the study involved *non-human primates*, add *additional details* about animal welfare and steps taken to ameliorate suffering<br>• If anesthesia, euthanasia, or any kind of animal sacrifice is part of the study, include briefly which substances and/or methods were applied<br><br>**Field Research**<br><br>Include the following details if this study involves the collection of plant, animal, or other materials from a natural setting:<br>• Field permit number<br>• Name of the institution or relevant body that granted permission	
**Data Availability**<br><br>Authors are required to make all data underlying the findings described fully available, without restriction, and from the time of publication. PLOS allows rare exceptions to address legal and ethical concerns. See the PLOS Data Policy and FAQ for detailed information.	No - some restrictions will apply

Powered by Editorial Manager® and ProduXion Manager® from Aries Systems Corporation

A Data Availability Statement describing where the data can be found is required at submission. Your answers to this question constitute the Data Availability Statement and **will be published in the article**, if accepted.<br><br>**Important:** Stating 'data available on request from the author' is not sufficient. If your data are only available upon request, select 'No' for the first question and explain your exceptional situation in the text box.<br><br>Do the authors confirm that all data underlying the findings described in their manuscript are fully available without restriction?	
**Describe where the data may be found in full sentences. If you are copying our sample text, replace any instances of XXX with the appropriate details.**<br><br>• If the data are **held or will be held in a public repository**, include URLs, accession numbers or DOIs. If this information will only be available after acceptance, indicate this by ticking the box below. For example: *All XXX files are available from the XXX database (accession number(s) XXX, XXX.).*<br>• If the data are all contained **within the manuscript and/or Supporting Information files**, enter the following: *All relevant data are within the manuscript and its Supporting Information files.*<br>• If neither of these applies but you are able to provide **details of access elsewhere**, with or without limitations, please do so. For example:<br><br>*Data cannot be shared publicly because of [XXX]. Data are available from the XXX Institutional Data Access / Ethics Committee (contact via XXX) for researchers who meet the criteria for access to confidential data.*<br><br>*The data underlying the results presented in the study are available*	Data cannot be shared publicly because of participants' privacy and confidentiality. The data underlying the results presented in the study are owned by University of Medicine and Pharmacy and are available upon requests.

from (include the name of the third party and contact information or URL).<br>• This text is appropriate if the data are owned by a third party and authors do not have permission to share the data.<br><br>* typeset	
Additional data availability information:	

Manuscript

Click here to access/download;Manuscript;Manuscript_PLOS ONE.docx

Full title: SOURCES OF STRESS, COPING STRATEGIES AND ASSOCIATED FACTORS AMONG VIETNAMESE FIRST-YEAR MEDICAL STUDENTS

Short title: Stress and coping strategies in Vietnamese medical students

Authors: Tan Nguyen[1], Christy Pu[1], Alexander Waits[1], Tuan D Tran[2], Yatan Pal Singh Balhara[3], Quynh Thi Vu Huynh[2], Song-Lih Huang[1*]

[1] Institute of Public Health, National Yang Ming Chiao Tung University, Taipei, Taiwan

[2] University of Medicine and Pharmacy, Ho Chi Minh City, Vietnam

[3] Department of Psychiatry, All India Institute of Medical Sciences (AIIMS), New Delhi, India

[*] Corresponding author: Song-Lih Huang

Email: songlih.huang@nycu.edu.tw

Abstract:

Objectives: This study aims to examine the sources of stress among first-year medical students; the frequency of their coping strategies; the factors associated with specific stressors and specific coping strategies adopted by the participants.

Methods: We conducted a cross-sectional study with 409 first-year students at the University of Medicine and Pharmacy, Vietnam. The survey comprised questions of socioeconomic status, stress-related issues, the six sources of stress (using Higher Education Stress Inventory), and the nine coping strategies (using Brief Coping Orientation to Problems Experienced). Factor analysis of HESI scores identified six sources of stress.

Results: Among the six sources of stress, “Worries about future competence/endurance” had the highest mean score (3.02±0.64), while “Mismatch in professional role expectations” had the lowest score (1.60±0.53). “Financial concerns” and “Academic workloads” were also significant sources of stress. Regarding coping strategies, Self-distraction was most frequently adopted by the participants (2.80 ± 0.68). Problem-solving (2.72±0.53) and seeking Social support (2.62±0.70) were also common adaptive strategies. Avoidance (1.87±0.55) and substance-use (1.27±0.55) were the least frequent strategies. Students who experienced acute stress event were more likely to have financial concerns compared to others. Substance use was positively associated with stressors from “Non-supportive educational environment”, “Having physical issues” and “Having part-time jobs”. Self-blame was more frequent among students with “Worries about future competence/endurance”, “Financial concerns”, “Academic workload”,

and “High perceived stress level”. Male student tended to adopt humor strategy (β=0.25), while less likely to utilize religious practices (β= -0.21, p=0.01).

Conclusions: Two-thirds of first-year medical students reported moderate to high levels of stress. “Worries about future competence/endurance” was the most concerned stressor, followed by “Academic workload”, and “Financial concerns”. The participants reported high frequency of utilization “Self-distraction”, “Problem-solving” and “Social support” when confronting stress. The findings may help inform the school management to better support students’ well-being.

Key words: sources of stress, coping strategies, medical students, Higher Education Stress Inventory, Brief COPE

I. Introduction

Stress and coping strategies have increasingly been studied, particularly in the population of medical students (1-3). Being exposed to prolonged and high levels of stress may lead to negative consequences: cognitive and emotional burden, psychological distress, dropping out of school, low quality of life and decreased empathy with patients, which all may result in medical errors and ~~may~~ influence the quality of health care service ~~in the wide aspect~~ (4-6). Research has suggested that medical students experience stress from different sources, which could be from academic factors, psychosocial factors with emphasis on financial issues, teacher-student relationships, and high expectations from significant others and self- (7, 8). Furthermore, in medical education settings, previous studies revealed that academic-related stressors may cause more distress than interpersonal, intrapersonal, or environmental stressors

(9). Early identification of these stressors could help tailor appropriate prevention and intervention programs to tackle the problematic psychological issues among this high-risk population.

Coping strategies are specific efforts, cognitively and behaviorally, that help a person to tolerate, to minimize or to handle stressors. When confronting stressors, a person may end up with different types of coping strategies depending on the primary appraisal of the stressor as challenging or threatening, then the second appraisal of their available resources to cope with stressors (10). There're different ways to categorize coping strategies, consisting of problem-focused or emotion-focused, adaptive or maladaptive, approaching or avoiding (11, 12). Adopting appropriate strategies would help students cope better and healthier with stressors, minimizing the negative consequences from stressors (13-15). Hence, it is essential to first understand what sources of stress that medical students are facing, then how they are coping with a variety of stressors. Furthermore, the link between potential associated factors with specific coping strategies would contribute to the understanding of coping model, which is important to figure out appropriate approaches to enhance the ability of students to deal effectively with stressors.

To date, few studies have directly examined how sociodemographic factors, or str[illegible]related factors are associated with sources of stressors and types of coping strategies. Our aims are to examine the sources of stress among first-year medical students, the frequencies of their coping strategies, and to explore the relationships between other factors with specific sources of stress and coping strategies employed by these students.

II. Methods

1. Study design:

We conducted a cross-sectional study among the undergraduate students at Faculty of Medicine, University of Medicine and Pharmacy, Ho Chi Minh City, Vietnam.

2. Participants:

In this study, we invited all first-year students. Participants provided written informed consents before participating in the study. Students with informed consents and completed data were included in the study with no explicit exclusion criteria were employed. There were 409 students completing the questionnaire, making the participation rate 97%.

3. Procedures:

Participants filled in a self-administered survey two months after school admission. The survey was anonymous with no information to identify the participants. Data were collected from 12th November 2020 to 22nd December 2020, and have been accessed for data analysis since 24th December 2020. The purpose of the study was explained to all participants, and instructions were given on how to fill in the online questionnaires, which consisted of four sections as mentioned below.

The survey included: (i) Demographic information (Age, Sex, Parental education status, Part-time job); (ii) Self-rated stress level, acute stress event, physical issues, psychological issues, Covid-19-related stress; (iii) Higher Education Stress Inventory (HESI) to assess the sources of

stress; (iv) Brief Coping Orientation to Problems Experienced (Brief COPE) to assess the coping strategies.

4. Measurement tools:

HESI

The original HESI was developed by Dahlin et al. in Sweden ~~(2005)~~ among medical students (16). The strength of this scale is its ability to assess various sources of stress in different higher education settings. From 33 items from the original version, we performed Exploratory Factor Analysis (EFA) and Confirmatory Factor Analysis (CFA) to get the revised scale with 20 items, categorized into six sources of stress. Cronbach's alpha was 0.73 for the whole scale. CMIN/DF was 1.97, CFI and RMSEA < 0.06, which indicates adequate degree of model fitting. It can be considered that the above extracted results are feasible and acceptable for this study population. Respondents rate each statement of these 20 items on a 4-point Likert~~-type~~ scale: 1 (Totally disagree) to 4 (Totally agree).

The six sources of stress included:

1. Mismatch in professional role expectations (from four items "The training demands that I join in situations that I find unethical"; "The professional role presented in the training conflicts with my personal view"; "I feel that I am less well treated because of my ethnic background"; and "I feel that I am less well treated because of my sex").

2. Worries about future competence/endurance (from three items "I worry about long working hours and responsibilities in my future career"; "The insight I have had into my future

profession has made me worries about the stressful workload"; "I am worried that I will not acquire all the knowledge needed for my future profession").

3. Financial concerns (from three items "As a student, my financial situation is a worry", "I am worried about my future economy and my ability to repay students loans"; "I am worried about accommodation").

4. Academic workload (from three items "The literature is too difficult and extensive"; "The space of studies is too high"; "Studies control my life and I have little time for other activities").

5. Low identity of medical profession (from three items "I am satisfied with my choice of career" – reversed score; "I am proud of my future profession" – reversed score; "I am able to influence my studies" – reversed score).

6. Non-supportive educational environment (from four items "Student union activities promote a sense of community and contribute to a better working environment for students" – reversed score; "The teachers often give feedback on students' knowledge and skills" – reversed score; "I feel that the training is preparing me well for my future profession" – reversed score; "My fellow students support me" – reversed score)

The score of each source of stress was the average score of their related items, ranging from 1.0 to 4.0.

BRIEF COPE

Brief COPE was developed as a short version of the original 60-item COPE scale, by Carver et al (1989) (17), (Carver, 1997) (18). Brief COPE is a 28-item measure, which is designed to assess

the frequency of utilization of various coping strategies on a scale of 1 (I haven't been doing this at all) to 4 (I've been doing this a lot). Our findings from EFA and CFA of the original Brief COPE revealed the revised 27-item scale with 9 categories of coping strategies. Cronbach's alpha was 0.78. CMIN/DF is 1.99, CFI is greater than 0.9, RMSEA < 0.06, and SRMR < 0.08 which indicates a good degree of model fitting, and the above extracted results are feasible and acceptable for this study population.

The nine coping strategies included:

1. Problem solving (from seven items "I've been taking action to try to make the situation better"; "I've been concentrating my efforts on doing something about the situation I'm in"; "I've been trying to come up with a strategy about what to do"; "I've been thinking hard about what steps to take"; "I've looking for something good in what is happening"; "I've been learning to live with it"; "I've been trying to see it in a different light, to make it seem more positive").

2. Social support (from four items "I've been getting help and advice from other people"; "I've been getting emotional support from others"; "I've been getting comfort and understanding from someone"; "I've been trying to get advice or help from other people about what to do").

3. Avoidance (from four items "I've been giving up the attempt to cope"; "I've been refusing to believe that it has happened"; "I've been giving up trying to deal with it"; "I've been saying to myself 'this isn't real'").

4. Substance use (from two items "I've been using alcohol or other drugs to make myself feel better"; "I've been using alcohol or other drugs to help me get through it").

5. Self-blame (from two items "I've been criticizing myself"; "I've been blaming myself for things that happened").

6. Religion (from two items "I've been praying or meditating"; "I've been trying to find comfort in my religion or spiritual beliefs")

7. Humor (from two items "I've been making fun of the situation"; "I've been making jokes about it").

8. Venting (from two items "I've been saying things to let my unpleasant feeling escape"; "I've been expressing my negative feelings").

9. Self-distraction (from two items "I've been doing something to think about it less, such as going to movies, watching TV, reading, daydreaming, sleeping or shopping"; "I've been turning to work or other activities to take my mind off things").

The score of each coping strategy was the average score of their related items, ranging from 1.0 to 4.0.

5. Analytical approach

The following five steps were taken to validate the Vietnamese versions of the HESI and Brief COPE: (1) translation, (2) assessment of content validity, (3) assessment of the factor structure relevant to medical students in this study, (4) assessment of internal consistency of the revised scales, (5) assessment of construct validity (EFA + CFA) of the revised scales.

We used RStudio (19) and SPSS 26 for data analyses. Descriptive statistics (mean, standard deviation, and percentage) were used for calculating the frequencies and proportions of

demographic variables, sources of stress, and coping strategies. To assess the factors associated with sources of stress and coping strategies, multiple linear regressions were estimated. For each source of stress, the regression model included all sociodemographic factors: sex, age, parental educational levels, part-time job, level of perceived stress, and other stress-related variables (acute stress event, physical stress, psychological stress, Covid-19-related stress). Similarly for each coping strategy, we run different regression models that included different sources of stress and all the above factors. Level of stress was scored from 1 to 3 for low to high level, respectively. Mother and father educational levels were categorized into three levels. Part-time job, acute stress event, physical stress, psychological stress, and Covid-19-related stress were binary variables with responses of yes or no.

This study was approved by the IRB of National Yang Ming Chiao Tung University and University of Medicine and Pharmacy, and guidelines for research with human subjects were followed.

III. Results

1. Sociodemographic of participants

Among the 409 participants, the mean age was 18.1 with a standard deviation of 0.38. Approximately 40% of participants were female. Most of the participants' parents finished their high school or higher. More than 20% of students reported experiencing either physical issues or psychological issues, while less than 10% had stress due to Covid-19. There were 231 students (57%) reported a moderate level of stress, and 36 students (8.9%) had a high level of stress.

Table 1: Characteristics of the study participants

	N = 409 students
Age	
Mean (SD)	18.1 (0.38)
Sex	
Female (N, %)	154 (38.2%)
Father education	
Primary school or lower (N, %)	11 (2.7%)
High school (N, %)	107 (26.6%)
College or higher (N, %)	285 (70.7%)
Mother education	
Primary school or lower (N, %)	21 (5.2%)
High school (N, %)	154 (38.2%)
College or higher (N, %)	228 (56.6%)
Part-time job	
Yes (N, %)	32 (7.9%)
Physical stress	
Yes (N, %)	98 (24.3%)
Psychosocial stress	
Yes (N, %)	92 (22.8%)
Covid-19 - related stress	
Yes (N, %)	39 (9.7%)

Stress level	
Low (N, %)	136 (33.7%)
Moderate (N, %)	231 (57.3%)
High (N, %)	36 (8.9%)

2. The sources of stress and the coping strategies adopted by the participants

The HESI subscale scores are listed in descending order from the participants. “Worries about future competence/endurance” had the highest mean score among 409 participants (3.02±0.64), while “Mismatch in professional role expectations” had the lowest score (1.60±0.53). “Financial concerns” and “Academic workloads” were also significant sources of stress among first-year students in this study, with the mean scores of 2.65 and 1.86, respectively. The other stressors with less concerns were “Low identity of medical profession” (mean score: 1.87) and “Non-supportive educational environment” (mean score: 1.80).

Table 2: Sources of stress among 409 students, using the revised 20-item HESI scale

**Sources of stress**	**Mean (SD)**
Worries about future competence/endurance	3.02 (0.64)
Academic workloads	2.87 (0.59)
Financial concerns	2.65 (0.75)
Low identity of medical profession	1.87 (0.52)
Non-supportive educational environment	1.80 (0.47)

Mismatch in professional role expectations	1.60 (0.53)

Regarding coping strategies employed by the study population, Self-distraction was the most frequently reported among these 409 students (2.80 ± 0.68). However, Problem-solving and seeking Social support were predominant the other less adaptive strategies, with the scores were 2.72 (0.53) and 2.62 (0.70), respectively. Self-blame also had a high score (2.52±0.74), i.e., it appears quite frequently among the participants. Avoidance (1.87±0.55) and substance-use (1.27±0.55) were the least frequent strategies adopted to cope with stress.

Table 3: Coping strategies scores of 409 students, using the revised 27-item Brief COPE scale

**Coping strategies**	**Mean (SD)**
Self-distraction	2.80 (0.68)
Problem solving	2.72 (0.53)
Social support	2.62 (0.70)
Self-blame	2.52 (0.74)
Venting	2.50 (0.70)
Humor	1.97 (0.79)
Religion	1.88 (0.81)
Avoidance	1.87 (0.55)
Substance use	1.27 (0.55)

3. Factors associated with sources of stress and coping strategies adopted by the participants.

Students who experienced acute stress event were more likely to have financial concerns compared to other students. On the other hand, "Low identity of medical profession" was found associated with female students and students who had part-time jobs.

Table 4: Factors associated with the six sources of stress

Factors	Estimates	p-value
**Financial concerns**		
Experiencing acute stress event	0.20	0.027
**Low identity of medical profession**		
Being male student	-0.14	0.007
Having part-time job	0.28	0.005

Only significant factors were listed in Table 4

Table 5 illustrated a variety of associated factors with coping strategies utilized by the participants. Students with "Low identity of medical profession" were less likely to seek social support (β=-0.25). Meanwhile, students with stressors due to "Mismatch in professional role expectations" tend to have both adaptive and maladaptive strategies: Humor (β=0.25), Religion (β=0.3), Avoidance (β=0.22), and Substance use (β=0.25). Substance use was also found positively associated with stressors from "Non-supportive educational environment", "Having physical issues" and "Having part-time jobs". On the other hand, Self-blame was reported higher frequency among students with "Worries about future competence/endurance"

(β=0.14), “Financial concerns” (β=0.17), “Academic workload” (β=0.18), “High perceived stress level” (β=0.20). Male student tent to adopt humor strategy (β=0.25, p=0.02), while less likely to utilize religious practices (β= -0.21, p=0.01).

Table 5: Factors associated with the nine coping strategies

**Factors**	**Estimates**	**p-value**
**Social support**		
Low identity of medical profession	-0.25	<0.001
**Avoidance**		
Mismatch in professional role expectations	0.22	<0.001
**Substance use**		
Mismatch in professional role expectations	0.25	<0.001
Non-supportive educational environment	0.15	0.010
Having physical issues	0.15	0.027
Having part-time job	0.20	0.048
**Self-blame**		
Worries about future competence/ endurance	0.14	0.013
Financial concerns	0.17	<0.001
Academic workload	0.18	0.003
Perceived stress level	0.20	0.002
**Religion**		

Mismatch in professional role expectations	0.30	<0.001
Being male student	-0.21	0.013
**Humor**		
Mismatch in professional role expectations	0.25	<0.001
Being male student	0.20	0.019

Only significant factors were listed in Table 5

IV. Discussions

The findings reveal that two-thirds of the participants rated their stress levels were moderate to high level after admission two months. This is comparable to other studies from Vietnam and other medical school in other countries. The study of Quynh et al. in 2020 showed almost 50% of students have problems with stress(20). Another study among 686 students found that 61.4% of the medical students reported different degrees of stress. However, only 2.4% of students rated their stress level as high in that study, whereas our finding showed 8.9% of the participants had high level of stress. Also in that study, the most prevalent sources of stress were from academic performance, including Test/exam (99% students), Falling behind in reading schedule (~97% students), Getting poor marks (~93% students), or Heavy workload (85.2% students). “Feeling of incompetence” was reported from 78.4%, while poor motivation to learn met in 48% of students (21). The sources of stress reported in our studies were measured by the revised 20-items HESI scale, which showed that Worries about future incompetence/endurance was the highest ranked stressor of the participants. “Academic workload” came as the second significant source of stress, followed by “Financial concerns”.

One remarkable observation was that the scores of the remaining three sources of stress were much lower than the first ranked three stressors above. This suggests that medical undergraduate students face a variety of stressors, which are not just limited to academic burden, but encompass their self-expectations and motivations to practice medical profession in the future.

Mismatch in professional role expectations with their inner conflicts to educational activities, served as other sources of stress. Pursuing medical practice requires students to understand patients' suffering and be willing to provide necessary treatment with professionalism and empathy. Accordingly, training activities would build up professional roles with specific expectations, including but not limited to being authentic, ethical, and respectful. Without the necessary supportive system, students would feel frustrated and stressed, particularly in their first year entering medical education. In our study, "Non-supportive educational environment" includes both the school environment and teacher-student relationships. Previous studies focus on students' experiences of homesick, difficulties in adapting new environments, as well as lacks of contact with family (22); or competitive, cold and impersonal attitudes (23). Since HESI was not designed specifically for medical education settings, we do not measure other sources of stress from medical training: facing illness or death of patients, parental wish for you to study medicine (9).

The role of gender when investigating stressors in medical students has long been studied, with inconsistent findings. In our study, female students and students were more likely to get stress due to their low identity of medical profession, but not other sources of stress. This finding was aligned with other studies as non-male gender has been identify as risk factor of medical

students' distress (24), particular stressor due to academic workload (25, 26). On the other hand, study of Yogesh et al of 100 first-year medical students showed that stress levels due to academic and interpersonal issues were lower in female compared to their male counterpart (7). Another study of Sadiq et al revealed higher levels of all stressors among female students, but no significant correlations between gender and sources of stress was found (9). Furthermore, our findings showed that students experiencing acute stress event tend to be stressed due to financial concerns, while students with part-time jobs were more likely to report stress of "Low identity of medical profession". We have not found any evidence of these social factors associated with stressors among medical students in the available research. Understanding the association of these socioeconomic factors and different sources of stress would help school administrators analyze and figure out appropriate approaches to support these students. Further studies may need to explore more these relationships.

Regarding coping strategies, Self-distraction was the most frequently used among 409 participants. However, active coping strategies (Problem-solving, seeking for Social support, Venting) were also popular in this study population. This was aligned with findings of other studies on coping strategies of medical students(27-29). On the other hand, Humor and Religion (Religious practice) were reported with lower frequency. ~~Religious practicing~~ was one of the most adopted coping strategies in some countries, but not in our study population. Positive religious practice has been proved positively effect on students' resilience and their mental health(30, 31). Among the maladaptive coping strategies, Self-blame had a relatively high score, whereas Substance-use and Avoidance were found not frequently adopted by the participants. These are positive findings among the first-year students since previous studies conducted in

the United Kingdom and in Nepal found “Substance use” as a very common coping strategies among medical students(32-34). However, under-reporting cannot be ruled out. Since substance-use was regarded as moral issue in Vietnam society, students might not be open about it despite the anonymity and confidentiality nature of our surveys.

Associations between different factors and coping strategies were observed. Overall, the strength of associations was low to moderate, yet in the expected directions. This study found that the levels of perceived stress was only linked to Self-blame, which was discrepant with ~~the~~ previous findings that high level of stress would increase the use of maladaptive coping strategies besides self-blame, including substance use, denial, wishful thinking, behavioral disengagement (35, 36). Male students were more likely to adopt humor strategy than their female counterpart, while less likely to have religious practice. This was similar to the results of a study conducted in a sample of 94 medical students in their third year about the use of humor strategy(37). This also added new insights into the previous studies about coping strategies’ differences among male and female medical students. Though the common findings were that female students tent to adopt emotional and instrumental support, venting and self-distraction (37, 38), we did not find the significant associations between students’ gender with these coping strategies.

Furthermore, we found that specific sources of stressors were linked to specific strategies when students confront stress. Either “Worries about future competence/endurance”, “Financial concerns” or “Academic workload” was found positively associated with Self-blame. On the other hand, when students had “Low identity of medical profession”, they were less likely to seek social support (emotional or instrumental support). While “Mismatch in professional role

expectations” was positively associated with the frequency of adoption Humor, Religion (Religious practice), Avoidance, and Substance use strategies; “Non-supportive educational environment” only significantly associated with Substance use among the participants. Finally, students with physical issues were more likely to use substances to deal with stress compared to those reported no stress due to physical issues. Provided that these associations between stressors and coping strategies have not been studied in the field of medical training, our findings were novel and would shed the light on approaches to enhance students’ coping strategies to deal with different sources of stress.

The study has some limitations, including the nature of cross-sectional study could not draw any causal relationship between the sources of stressors and coping strategies adopted by the participants. Also, when measuring the frequency of coping strategies, the questions did not specify the sources of stress. Hence, we could not imply the high frequency of any coping strategy due to any specific source of stressors. However, the strength of this study was the participation of 97% of first-year students in a school, enhancing the representative of the study population. Furthermore, the instruments used were validated in this study population, ensuring the validity and reliability of the scales.

V. Conclusions

Medical students were exposed to a variety of stressors since their first year of training, which resulted in two-thirds of students reporting moderate to high level of stress in this study. “Worries about future competence/endurance” was the most concerned stressor, followed by “Academic workload”, and “Financial concerns”. The participants reported high frequency of

utilization "Self-distraction", "Problem-solving" and seeking "Social support" when dealing with stress. The findings revealed significant associated factors of sources of stress and coping strategies adopted by first-year medical students. These observations may help inform the school administrators and leaders of the status of stressors and coping as well as suggest appropriate approaches to support their students' well-being.

Acknowledgements

We would like to thank our participants for their willingness to complete the surveys. We also would like to express our gratitude to the school leaders as being supportive for us to accomplish this study.

References

1. Yusoff MS, Abdul Rahim AF, Yaacob MJ. Prevalence and Sources of Stress among Universiti Sains Malaysia Medical Students. The Malaysian journal of medical sciences : MJMS. 2010;17(1):30-7.
2. Melaku L, Bulcha G. Evaluation and Comparison of Medical Students Stressors and Coping Strategies among Undergraduate Preclinical and Clinical Year Students Enrolled in Medical School of Arsi University, Southeast Ethiopia. Educ Res Int. 2021;2021:9202156.
3. Mosley TH, Jr., Perrin SG, Neral SM, Dubbert PM, Grothues CA, Pinto BM. Stress, coping, and well-being among third-year medical students. Academic medicine : journal of the Association of American Medical Colleges. 1994;69(9):765-7.
4. Ruzhenkova V, Ruzhenkov V, Khamskaya I, Anisimova NA. Academic stress and its effect on medical students' mental health status. Drug Invention Today. 2018;10:1171-4.

5. Gleichgerrcht E, Decety J. Empathy in Clinical Practice: How Individual Dispositions, Gender, and Experience Moderate Empathic Concern, Burnout, and Emotional Distress in Physicians. PLoS One. 2013;8(4):e61526.

6. Dyrbye LN, Harper W, Durning SJ, Moutier C, Thomas MR, Massie FS, Jr., et al. Patterns of distress in US medical students. Medical teacher. 2011;33(10):834-9.

7. Saxena Y, Shrivastava A, Singhi P. Gender correlation of stress levels and sources of stress among first year students in a medical college. Indian journal of physiology and pharmacology. 2014;58:147-51.

8. Ragab EA, Dafallah MA, Salih MH, Osman WN, Osman M, Miskeen E, et al. Stress and its correlates among medical students in six medical colleges: an attempt to understand the current situation. Middle East Current Psychiatry. 2021;28(1):75.

9. Sadiq A, Ashraf MF. Measuring the Stressors in Undergraduate Medical Students: A Cross Sectional Study. Sustainable Business and Society in Emerging Economies. 2022;3:367-73.

10. Frydenberg E. Coping research: Historical background, links with emotion, and new research directions on adaptive processes. Australian Journal of Psychology. 2014;66(2):82-92.

11. Minchekar V. Effect of Problem-Focused and Emotion-Focused Coping Strategies on Academic Stress during Examinations. 2017;6:57-9.

12. García FE, Barraza-Peña CG, Wlodarczyk A, Alvear-Carrasco M, Reyes-Reyes A. Psychometric properties of the Brief-COPE for the evaluation of coping strategies in the Chilean population. Psicologia: Reflexão e Crítica. 2018;31.

13. Yusoff MSB, Yee LY, Wei LH, Siong TC, Meng LH, Bin LX, et al. A study on stress, stressors and coping strategies among Malaysian medical students. 2011;1(2).

14. Kasi PM, Khawar T, Khan FH, Kiani JG, Khan UZ, Khan HM, et al. Studying the association between postgraduate trainees' work hours, stress and the use of maladaptive coping strategies. Journal of Ayub Medical College, Abbottabad : JAMC. 2007;19(3):37-41.

15. Shoua-Desmarais N, von Harscher H, Rivera M, Felix T, Havas N, Rodriguez P, et al. First Year Burnout and Coping in One US Medical School. Academic psychiatry : the journal of the American Association of Directors of Psychiatric Residency Training and the Association for Academic Psychiatry. 2020;44(4):394-8.

16. Dahlin M, Joneborg N, Runeson B. Stress and depression among medical students: a cross-sectional study. Medical education. 2005;39(6):594-604.

17. Carver CS,

Scheier, M. F.,

Weintraub, J. K. COPE Inventory [Database record]. APA PsycTests. 1989.

18. Carver CS. You want to measure coping but your protocol's too long: consider the brief COPE. International journal of behavioral medicine. 1997;4(1):92-100.

19. Team R. RStudio: Integrated Development Environment for R. RStudio, PBC, Boston, MA 2022.

20. Huynh Q, Tanasugarn C, Kengganpanich M, Lapvongwatana P, Quynh Long K, Thai TT. Mental Well-being, and Coping Strategies during Stress for Preclinical Medical Students in Vietnam. Journal of Population and Social Studies. 2019;28:116-29.

21. Saipanish R. Stress among medical students in a Thai medical school. Medical teacher. 2003;25(5):502-6.

22. Qamar K, Khan N, Kiani MR. Factors associated with stress among medical students. JPMA The Journal of the Pakistan Medical Association. 2015;65:753-5.

23. Shim E-J, Jeon HJ, Kim H, Lee K-M, Jung D, Noh H-L, et al. Measuring stress in medical education: validation of the Korean version of the higher education stress inventory with medical students. BMC medical education. 2016;16.

24. Rajapuram N, Langness S, Marshall MR, Sammann A. Medical students in distress: The impact of gender, race, debt, and disability. PLoS One. 2020;15(12):e0243250.

25. Backović DV, Zivojinović JI, Maksimović J, Maksimović M. Gender differences in academic stress and burnout among medical students in final years of education. Psychiatria Danubina. 2012;24(2):175-81.

26. Pushpanathan P, Saranya K, Chandrasekar.M, Subramanian S. EFFECT OF GENDER ON SIX DOMAINS OF STRESS IN MEDICAL STUDENTS. International Journal of Current Research. 2016;8:26938-44.

27. Neufeld A, Malin G. How medical students cope with stress: a cross-sectional look at strategies and their sociodemographic antecedents. BMC medical education. 2021;21(1):299.

28. Bamuhair SS, Al Farhan AI, Althubaiti A, Agha S, Rahman Su, Ibrahim NO. Sources of Stress and Coping Strategies among Undergraduate Medical Students Enrolled in a Problem-Based Learning Curriculum. Journal of Biomedical Education. 2015;2015:575139.

29. Pitanupong J. Stress and Coping Strategies among Thai Medical Students in a Southern Medical School. Siriraj Medical Journal. 2020;72:238-44.

30. Rezapur-Shahkolai F, Taheri-Kharameh Z, Moeini B, Khoshravesh S. Religious coping and resilience among students of medical sciences: Forouzan Rezapur Shahkolai. European Journal of Public Health. 2017;27(suppl_3):ckx187.354.

31. Francis B, Gill JS, Yit Han N, Petrus CF, Azhar FL, Ahmad Sabki Z, et al. Religious Coping, Religiosity, Depression and Anxiety among Medical Students in a Multi-Religious Setting. International journal of environmental research and public health. 2019;16(2).

32. Ashton CH, Kamali F. Personality, lifestyles, alcohol and drug consumption in a sample of British medical students. Medical education. 1995;29(3):187-92.

33. Shyangwa PM, Joshi D, Lal R. Alcohols and other substance use/abuse among junior doctors and medical students in a teaching institute. JNMA J Nepal Med Assoc. 2007;46(167):126-9.

34. Zvauya R, Oyebode F, Day EJ, Thomas CP, Jones LA. A comparison of stress levels, coping styles and psychological morbidity between graduate-entry and traditional undergraduate medical students during the first 2 years at a UK medical school. BMC research notes. 2017;10(1):93.

35. Garbóczy S, Szemán-Nagy A, Ahmad MS, Harsányi S, Ocsenás D, Rekenyi V, et al. Health anxiety, perceived stress, and coping styles in the shadow of the COVID-19. BMC psychology. 2021;9(1):53.

36. Kumanova M, Karastoyanov G. Perceived Stress and Coping Strategies2013.

37. Madhyastha S, Latha KS, Kamath A. Stress, Coping and Gender Differences in Third Year Medical Students. Journal of Health Management. 2014;16(2):315-26.

38. Graves BS, Hall ME, Dias-Karch C, Haischer MH, Apter C. Gender differences in perceived stress and coping among college students. PLoS One. 2021;16(8):e0255634.

Click here to access/download
Supporting Information
STROBE-checklist-v4-combined-PlosMedicine_15195.docx
