# Supplementary material for: Sources of stress, coping strategies and associated factors among Vietnamese first-year medical students
Source: PLoS One. 2024 Jul 31;19(7):e0308239. doi: 10.1371/journal.pone.0308239 (PMC11290621; doi:10.1371/journal.pone.0308239)
Supplement: Attachment — Submitted filename: PlosOne_Response to Reviewers and Editor_11.09.docx [file pone.0308239.s003.docx]

**Reviewers’ comments**

Dear Reviewers,

We are grateful for your time and effort reviewing our manuscript. Your comments and suggestions directed us to improve our analysis and writing. Kindly refer to the point-by-point revisions we conducted in response to your comments.

**Reviewer 1**

Dear reviewer, we are very grateful for your time dedicated to reading our manuscript. Your comments provided multiple insights and helped us to improve our writing. We tried our best to carefully address your comments. Kindly refer to the details below.

**1. I understand the sources of external stressors which cause difficulty of quality of life index for medical students. However, it's equally important to indicate the physiological stressors as well. Two recent articles demonstrated how a physiological abnormality like PMS/PMDD may lead to low academic and social life of medical students leading to additional amount of internal stressors. It will be beneficial for our reader and the policy makers at least to be aware of this too. Here are the two links:
DOI: 10.7759/cureus.40141
DOI: 10.3389/fmed.2022.821908**

We added the statement and the discussion of the effects of physical issues or physiological stressors on medical students’ academic performance and quality of life, using the references from your recommendation (Line 55-57, Line 300-302, and Line 305-307 in the revised manuscript) in the Introduction section and in the Discussion section, as following:

- In Introduction section:

“In addition, physical issues could also be an important stressor for medical students, which affects their academic performance and quality of life.”

- In Discussion section:

“Previous studies also suggested that psychosomatic discomfort, e.g., menstrual disturbance or polycystic ovary syndrome in female medical students, had negative effects on students’ academic performance and their quality of life.”

“However, in this study we utilized the HESI instrument that could not examine the physiological stressors of medical students and the associations of these stressors with coping strategies adopted by medical students.”

**2. Results: Overall comments: The results are expressed in table format which is fine for me however, please use graphical format for better presentation and use the table as supplemental as required. My intention is not to make you work more but to make your manuscript more readable.**

**Table 1: Characteristics of the study participants. This table can be easily shown with a bar diagram/scatter plot or by any visual graphical way. Therefore, represent the data of table 1 with a graph and attach the table as supplemental if necessary.**

**Table 2: Sources of stress among 409 students, using the revised 20-item HESI scale. This table has the mean and SD values. Therefore, please use a graph rather than the table to represent the data.
Table 3: Coping strategies scores of 409 students, using the revised 27-item Brief COPE scale. Please do the same for this table as well.
Table 4: Factors associated with the six sources of stress. This table may stay as it is.
Table 5: Factors associated with the nine coping strategies. As per this table some of the factors such as social support has p value of <0.001 whereas the other has >0.001. Therefore, use the data of this table to convert to a graphical chart(may be a bar diagram) and then indicate which bars are significant by adding * on top that bar. I hope this makes sense. This is the traditional way of representing data. Off course, you can add the table as supplemental if required.**

Thank you for your thorough suggestions and instructions on the use of figures in our manuscript. We have added Figure 1 and Figure 2 to replace Table 2 and Table 3 in the old manuscript. For Table 1, however, we’d like to keep the table format to show detailed information about demographic factors of the participants. Similarly, for Table 5, we’d like to keep as it is to have the consistency of data presentation from the factors associated with stressors and with coping strategies.

**3. Discussion: The discussion is well written however please find my suggestions below.
Generally, the discussion starts with a paragraph stating the problem, hypothesis. However, the authors directly started discussion the result. This disrupts the flow and readability. Please modify.**
We added a paragraph in the Discussion section to state the objectives and our approaches (Line 264-279 in the revised manuscript), as following:

“This study examined the sources of stress and the coping strategies adopted by first-year medical students at University of Medicine and Pharmacy at Ho Chi Minh City, Vietnam. Analyses were conducted to investigate whether there are associations between specific sources of stress and specific coping strategies. We found that specific sources of stressors were linked to specific strategies when students confront stress. Either “Worries about future competence/endurance”, “Financial concerns” or “Academic workload” was found positively associated with Self-blame. On the other hand, when students had “Low identity of medical profession”, they were less likely to seek social support (emotional or instrumental support). While “Mismatch in professional role expectations” was positively associated with the likelihood of adoption different coping strategies, namely Humor, Religion (Religious practice), Avoidance, and Substance use strategies; the source of stress named “Non-supportive educational environment” was only significantly associated with Substance use among the participants. Moreover, students with physical issues were more likely to use substances to deal with stress compared to those reported no stress due to physical issues. These associations were found independent of demographic factors, psychosocial factors and stress-related factors.” **The discussion section should include references of systematic review and meta-analysis of medical student and stressor. You may consider using these recent references
DOI: 10.7759/cureus.40141
DOI: 10.3389/fmed.2022.821908**

We added two sentences in the Discussion section to mention the effects of physiological issues on medical students’ academic performance and quality of life as you suggested, and why we did not measure the physiological stressors of medical students (Line 300-302, and Line 305-307 in the revised manuscript), as following:

“Previous studies also suggested that psychosomatic discomfort, e.g., menstrual disturbance or polycystic ovary syndrome in female medical students, had negative effects on students’ academic performance and their quality of life.”

“However, in this study we utilized the HESI instrument that could not examine the physiological stressors of medical students and the associations of these stressors with coping strategies adopted by medical students.”

**Reviewer 2**

Dear reviewer, we are very grateful for your time dedicated to reading our manuscript. Your comments provided multiple insights and helped us to improve our writing. We tried our best to carefully address your comments. Kindly refer to the details that we’ve revised based on your comments and suggestions from the PDF file.

In summary, there’re some points that we’ve revised to elaborate more details:

- The objectives of this study include examining the associations between specific sources of stress and specific coping strategies. Although there have been multiple studies on the associations between demographic factors and psychosocial factors with sources of stress or with coping strategies. To the best of our knowledge, the associations between these two main outcomes have not been investigated. We revised the statement to be clearer (Line 79-81 and Line 226-229 in the revised manuscript), as following:
  “To date, few studies have directly examined the associations between specific sources of stress and types of coping strategies.”
- We added a brief description of the university and the medical program as you suggested (Line 76-79 in the revised manuscript), as following:

“The University of Medicine and Pharmacy (UMP) in Ho Chi Minh City has adopted a reformed medical program, which is outcome-based medical education. This reformed program was considered with higher demands and more stressful than the old program, particularly for first-year students who have just entered medical training.”

- We elaborated our approaches to collect data (Line 93 and Line 99-105 in the revised manuscript), as following:

“Amongst 420 students, 409 students participated in this study and completed the questionnaire.”

“The principal investigator introduced the purpose of the study to all first-year students in an orientation at the beginning of the academic year. At the end of the orientation, the students sent back the consent forms to members of the research team. We sent a link of the survey to all students with written informed consent. The online survey was designed so all questions need to be completed before submission. The instructions were also given on how to fill in the online questionnaires,”

- We moved the variable definitions from the Analytical approach section to Procedure section as you suggested, (Line 110-114 in the revised manuscript), as following:
  “Level of stress was scored from 1 to 3 for low to high level, respectively. Mother and father educational levels were categorized into three levels (Elementary or lower, High school, and College or higher). Part-time job, acute stress event, physical stress, psychological stress, and Covid-19-related stress were binary variables with responses of yes or no.”
- We moved the description of instruments’ validation steps from the Analytical approach section to Measurement tools section as you suggested. We also add elaboration on how the items of the questionnaires were modified throughout five-step validation process (Line 116-125 in the revised manuscript), as following:

“The HESI and Brief COPE went through five steps of validation including: (i) Forward translation, (ii) Backward translation, (iii) Assessment of content validity, (iv) Assessment of factor structure, and (v) Assessment of internal consistency. During the first step, the English instruments were translated from English to Vietnamese separately by two translators (the principal investigator and another team member who has the bachelors in both psychology and English. The second step was conducted by two Vietnamese who has lived in the United state for than 10 years and used English in their daily work. Any ambiguities or discrepancies in terms of context meaning or colloquialism were discussed and resolved through consensus among research team members. The results of the fourth and fifth steps are elaborated as following:”

- We added the statement that the survey was designed to have all the items completed before submission, so we did not have missing data from our dataset (Line 103-104 in the revised manuscript), as following:

“The online survey was designed so all questions need to be completed before submission.”

- We revised Table 1 for the wrong numbers in the table. This was because we mistakenly keyed in from the outputs of statistical software. Thank you very much for your revisions.
- We added references, elaborated the references, and revised the writing in the Discussion part regarding the prevalence of stress among medical students (Line 282-294 in the revised manuscript), as following:

“A cross-sectional study conducted among 411 first-year students at the University of Medicine of Pharmacy of Ho Chi Minh City and Can Tho in 2020 showed almost 50% of students have problems with stress. Another cross-sectional study in 2003 among 686 students at the Faculty of Medicine, at Ramathibodi Hospital in Thailand found that 61.4% of the medical students reported different perceived levels of stress. However, only 2.4% of students rated their stress level as high in that study, whereas our finding showed 8.9% of the participants had high level of stress. Also in the above study, when rating the sources of stress from a questionnaire, the most prevalent sources of stress of these 686 medical students were from academic performance, including Test/exam (99% of the students), Falling behind in reading schedule (~97% of the students), Getting poor marks (~93% of the students), or Heavy workload (85.2% of the students). “Feeling of incompetence” was reported from 78.4%, while poor motivation to learn met in 48% of the students”

- We added references, and revised the writing in the Discussion part regarding then religious practice of medical students (Line 343-345 in the revised manuscript), as following:

“Religious practicing was one of the most adopted coping strategies in some countries (32,33), but not in our study population. Positive religious practice has been proved to have positive effect on students’ resilience and their mental health.”

- Besides the above, we’ve revised the typo, rewrote the manuscript, added details in the revised manuscript, along with your comments and suggestions, as you may find:
  - Line 272-276 in the revised manuscript, as following:

“While “Mismatch in professional role expectations” was positively associated with the likelihood of adoption different coping strategies, namely Humor, Religion (Religious practice), Avoidance, and Substance use strategies; the source of stress named “Non-supportive educational environment” was only significantly associated with Substance use among the participants.”

- Line 372-374 in the revised manuscript, as following:

“Also, when using the Brief COPE to measure the frequency of coping strategies adopted by medical students, the items did not specify the sources of stress that result in such coping strategies.”

- Line 375-380 in the revised manuscript, as following:

“However, the strength of this study was the participation of 97% of first-year students in a school, enhancing the representative of the first-year medical students at Faculty of Medicine, at University of Medicine and Pharmacy. Furthermore, the instruments used went through the five-step validation among first-year medical students, ensuring the validity and reliability of the scales.”

- Line 382-384 in the revised manuscript), as following:

“Medical students were exposed to a variety of stressors since their first year of training, and the results showed that two-thirds of students reporting moderate to high level of stress.”

- We’ve checked the Reference to make sure it follows the format of the journal, which is Vancouver style.
