# Supplementary material for: Sources of stress, coping strategies and associated factors among Vietnamese first-year medical students
Source: PLoS One. 2024 Jul 31;19(7):e0308239. doi: 10.1371/journal.pone.0308239 (PMC11290621; doi:10.1371/journal.pone.0308239)
Supplement: Attachment — Submitted filename: PlosOne_Response to Reviewers and Editor_25.03.docx [file pone.0308239.s004.docx]

**Reviewers’ comments**

Dear Reviewers,

We are grateful for your time and effort reviewing our manuscript. Your comments and suggestions directed us to improve our analysis and writing. Kindly refer to the point-by-point revisions we conducted in response to your comments.

**Reviewer 1**

Dear reviewer, we are very grateful for your time dedicated to reading our manuscript. Kindly refer to the details below.

**1. I have one very minor request. Please improve the quality of the figure 1 and 2. I think there are ways to improve the image quality. The written part of the figures are not clearly visible.**

We have already uploaded the new versions of Figure 1 and Figure 2, using the tool provided in the instruction.

**Reviewer 2**

Dear reviewer, we are very grateful for your time dedicated to reading our manuscript. Kindly refer to the details that we’ve revised based on your comments and suggestions from the PDF file.

**1. The authors mentioned that the university has adopted a new reform medical program and that is a more stressful for the students.
P 14
‘’The University of Medicine and Pharmacy (UMP) in Ho Chi Minh City has adopted a reformed medical program, which is outcome-based medical education. This reformed program was considered with higher demands and more stressful than the old program, particularly for first-year students who have just entered medical training.’’
Please describe the characteristics of the program, why do you consider it more stressful, what is the difference with the previous program? Do you have any evidence for this? And why the first-year students are affected, did the change was in the middle of university year? Otherwise the current program is the only thing they no directly.**

We have elaborated more details of the reformed program and explain why we perceived it more stressful for medical students, particularly for first-year students. Please refer to Line 77 – Line 86 of the revised manuscript: (We also added the reference of the reformed program of the university from 2016 – reference 18)

This reformed program was considered with higher demands than the old program. Students were introduced to family- and community-based medicine in the early phase of education. New components were integrated in the curriculum, namely Professionalism, Interprofessional education, Scholarly project, and Practice of medicine with the purpose of training students on basic clinical skills in the first two years. A student-centered approach was adopted in all teaching and learning settings ^1^. The self-directed learning requires students to be more proactive, which is stressful for students who are not familiar with this new education method, particularly the first-year students who have just entered medical training.

**2. The study was carried out in 2020, the students were online or onsite? As it was during the pandemic.**

During the time we collected these surveys, the students still studied at the university (onsite). The study was conducted before the surge in 2021, when we had a strict quarantine policy.

**3. The references are not in Vancouver style as required by the Journal.
Reference 15 missing the Journal and reference 19 either is missing the Journal or it is not a paper and should be quoted differently.**

We have revised the references following the Vancouver style and added the Journal of reference 15 and reference 19, which has become reference 20.

1.	Tran TD, Vu PM, Pham HTM, et al. Transforming medical education to strengthen the health professional training in Viet Nam: A case study. *The Lancet regional health Western Pacific*. Oct 2022;27:100543. doi:10.1016/j.lanwpc.2022.100543
