# Supplementary material for: Sources of stress, coping strategies and associated factors among Vietnamese first-year medical students
Source: PLoS One. 2024 Jul 31;19(7):e0308239. doi: 10.1371/journal.pone.0308239 (PMC11290621; doi:10.1371/journal.pone.0308239)
Supplement: Attachment — Submitted filename: Ressponses to Reviewers_27.06.docx [file pone.0308239.s005.docx]

Dear Reviewers,

We are grateful for your time and effort reviewing our manuscript. Your comments and suggestions directed us to improve our analysis and writing. Kindly refer to the point-by-point revisions we conducted in response to your comments.

**Reviewer 4**:

**The authors discuss the analysis results of this study, suggesting that the newly introduced outcome-based medical education program may be a contributing factor to stress among medical students. However, as there is no comparison with data from before the new program was introduced, the discussion appears speculative. Please remove statements that may lead to misinterpretation. Additionally, please consider citing studies on stress levels among medical students in other countries and discuss whether stress levels among medical students in Vietnam are particularly high.**

- Thank you for your opinion and recommendations. We have removed the description of the transformed outcome-based medical education program of the university. We also added the statement and references of the situation of stress among medical students in Vietnam (Line 48 – 50 in the revised manuscript) in the Introduction section as following:

“Studies in Vietnam show that the prevalence of experiencing stress among medical students are high, with more than 30% of the students perceived moderate to high level of stress.”

- We added a statement to elaborate the rationale to conduct this study among first year students (Line 80 in the revised manuscript) in the Introduction section as following:

“To date, few studies have directly examined the associations between specific sources of stress and types of coping strategies, particularly in the early phase of medical training.”

**In this study, multivariate analysis was conducted using multiple regression analysis. However, the authors' description does not ensure reproducibility. Moreover, only partial results are presented in the tables. Please clearly specify the dependent variables and the independent variables included for each test. It is also not mentioned whether forced entry or stepwise methods were used for independent variable selection.**

Thank you for your question to clarify this part. We provided more details about data analysis approaches as your recommendations (Line 203 – 209 in the revised manuscript) in the Analytical approach as following:

“To assess the factors associated with sources of stress and coping strategies, multiple linear regressions, with forced entry regressions, were estimated. For each source of stress as dependent variable, the independent variables included all sociodemographic factors: sex, age, parental educational levels, part-time job, level of perceived stress, and other stress-related variables (acute stress event, physical stress, psychological stress, Covid-19-related stress). Similarly for each coping strategy as dependent variable, we run different regression models that included different sources of stress and all the above sociodemographic and stress-related factors as independent variables.”

**As per my understanding, it seems that the authors conducted 15 repeated multiple regression analyses, with sociodemographic characteristics (age, sex, father education, mother education, part-time-job, physical stress, psychosocial stress, COVID-19 related stress, stress level) as independent variables, and a total of 15 items from HESI and Brief COPE as dependent variables. This testing method appears to have significant flaws. Since each item of HESI and Brief COPE uses a Likert-type scoring method, they are categorical variables rather than continuous variables, making their use as dependent variables in multiple regression analysis methodologically incorrect. It would be more appropriate to conduct multinomial logistic regression analyses for each item of HESI and Brief COPE to calculate odds ratios. Additionally, correction for multiple testing should be applied. Please conduct a fundamental review of the methodology.**

- Thank you for your recommendation. We have reviewed the analysis and revised the analysis, basically on the types of the independent variables (changed the type of Father educational level, Mother educational level, and Perceived stress levels into Categorical variables instead of Continuous variables as in the previous analysis). We have revised the results in Table 2 and Table 3 in the revised manuscripts, as following:

Table 2: Factors associated with the six sources of stress

| Factors | Estimates | p-value |
| --- | --- | --- |
| Mismatch in professional role expectations |  |  |
| Low perceived stress level | -0.32 | 0.003 |
| Worries about future competence/endurance |  |  |
| Being male student | -0.23 | <0.001 |
| Low perceived stress level | -0.28 | 0.033 |
| Financial concerns |  |  |
| Experiencing acute stress event | 0.20 | 0.027 |
| Low identity of medical profession |  |  |
| Being male student | -0.14 | 0.007 |
| Having part-time job | 0.28 | 0.005 |
| Non-supportive educational environment |  |  |
| Low perceived stress level | -0.28 | 0.004 |
| Moderate perceived stress level | -0.19 | 0.030 |

*For each source of stress, we ran a separate regression. The regression models were adjusted for: sex, age, parental educational levels, part-time job, level of perceived stress, acute stress event, physical stress, psychological stress, Covid-19-related stress.*

*Only factors with statistical significance were listed in Table 2.*

Table 3: Factors associated with the nine coping strategies

| Factors | Estimates | p-value |
| --- | --- | --- |
| Problem solving |  |  |
| Low identity of medical profession | -0.11 | 0.029 |
| Social support |  |  |
| Low identity of medical profession | -0.25 | <0.001 |
| Avoidance |  |  |
| Mismatch in professional role expectations | 0.22 | <0.001 |
| Substance use |  |  |
| Mismatch in professional role expectations | 0.25 | <0.001 |
| Non-supportive educational environment | 0.15 | 0.011 |
| Having physical issues | 0.15 | 0.033 |
| Having part-time job | 0.20 | 0.048 |
| Self-blame |  |  |
| Worries about future competence/ endurance | 0.14 | 0.013 |
| Financial concerns | 0.17 | <0.001 |
| Academic workload | 0.18 | 0.003 |
| Religion |  |  |
| Mismatch in professional role expectations | 0.30 | <0.001 |
| Being male student | -0.21 | 0.013 |
| Humor |  |  |
| Mismatch in professional role expectations | 0.25 | <0.001 |
| Being male student | 0.19 | 0.020 |
| Self-distraction |  |  |
| Having psychological issues | -0.23 | 0.013 |

*For each coping strategy, we ran a separate regression. The regression models were adjusted for: sources of stress, sex, age, parental educational levels, part-time job, level of perceived stress, acute stress event, physical stress, psychological stress, Covid-19-related stress.*

*Only factors with statistical significance were listed in Table 3.*

- We added the elaboration of the findings in Table 2 and Table 3 (Line 245 – 251, Line 258 – 259 and Line 267 – 270 in the revised manuscript) in the Results section as following:

“Students who reported high perceived stress level are more likely to get stressed due to “Mismatch in professional role expectations”, “Worries about future competence/endurance”, and “Non-supportive educational environment”. Students experiencing acute stress event were more likely to have financial concerns compared to other students (β=0.20). On the other hand, “Low identity of medical profession” was found associated with female students and students who had part-time jobs. Female students are also more likely to have “Worries about future competence/endurance”.”

“Table 3 illustrates a variety of associated factors with coping strategies utilized by the participants. Students with “Low identity of medical profession” were less likely to have orientations to solve the problem (β=-0.11) or to seek social support (β=-0.25).”

“Male student tent to adopt humor strategy (β=0.19, p=0.020), while less likely to utilize religious practices (β=-0.21, p=0.013). Meanwhile, students experiencing psychological issues tend to not use “Self-distraction” as a coping orientation to their problems (β= -0.23, p=0.013).”

- Regarding the statistical characteristics of the dependent variables (six sources of stress and nine coping strategies), please refer to the following elaboration:

Although items in the two tools Higher Education Stress Inventory (HESI) and Brief Coping Orientation to Problems Experienced (Brief COPE), were scored using the Likert-type scales, they are typically treated as scores rather than categories, and analyzed based on the summation of scores from clusters of items rather than on the score of an individual item. For HESI, the higher scores indicate higher levels of perceived stressor (1-3). Similarly, for Brief COPE, the higher scores indicate the more frequently the coping orientations were adopted (4-6). In this study, we analyzed the data as previous studies. We conducted analysis to examine the level of perceived stressors, the frequency of adopting coping orientations, and the association (if any) between the perceived stressors and the coping orientations.

Following your recommendations, we’ve performed the binominal logistic regression, in which each factor of HESI and each factor of Brief COPE were treated as binary variable. To be more specific, for HESI, each factor has two categories as Yes (I agree I have this stressor) and No (I disagree I have this stressor). For Brief COPE, each factor has two categories as Yes (I usually adopt this coping orientation to experienced problems) and No (I do not usually adopt this coping orientation to experienced problems).

We have run the Binominal Logistic Regressions and please refer to the findings in Appendix enclosed to this Response, but we have opted to present the data in the same manner as previous studies (1-6).

**The authors mention the high participation rate of the study participants (97%) as a strength of the study. However, we are concerned that this high participation rate may reflect a distortion of participants' voluntary consent. Please clarify the relationship between the authors and the students. Would you say there is any possibility at all that vulnerable students were coerced into participating in the study? Explain the ethical concerns.**

This study is among the three studies of a project to provide the Transforming Stress Program to all first-year students. This Transforming Stress Program was introduced to all first-year students in their orientation session at the beginning of the school year. It was introduced as an extra-curricular activity, and there was no other measure taken to enhance participation. The fact that it was endorsed by the school administration may help explain the high participation rate. Before they took the training, we invited them to fill out the survey to have the baseline information about their current stress mindset, stressors, and coping strategies on a voluntary basis. The process involves nothing of a coercive nature.

The Principal Investigator was an alumnus of the University and has no relationship with the first-year students. This study had been approved by the IRB of the University. We introduced the study to all students and got their agreement to join in this study by the written Informed Consent Forms.

1.	Saxena SK, Mani RN, Dwivedi AK, Ryali V, Timothy A. Association of educational stress with depression, anxiety, and substance use among medical and engineering undergraduates in India. Industrial psychiatry journal. 2019;28(2):160-9.

2.	Pacheco JPG, Hoffmann MS, Braun LE, Medeiros IP, Casarotto D, Hauck S, et al. Translation, cultural adaptation, and validation of the Brazilian Portuguese version of the Higher Education Stress Inventory (HESI-Br). Trends in psychiatry and psychotherapy. 2023;45:e20210300.

3.	Shim E-J, Jeon HJ, Kim H, Lee K-M, Jung D, Noh H-L, et al. Measuring stress in medical education: validation of the Korean version of the higher education stress inventory with medical students. BMC Medical Education. 2016;16.

4.	Matsumoto S, Yamaoka K, Nguyen HDT, Nguyen DT, Nagai M, Tanuma J, et al. Validation of the Brief Coping Orientation to Problem Experienced (Brief COPE) inventory in people living with HIV/AIDS in Vietnam. Global health & medicine. 2020;2(6):374-83.

5.	García FE, Barraza-Peña CG, Wlodarczyk A, Alvear-Carrasco M, Reyes-Reyes A. Psychometric properties of the Brief-COPE for the evaluation of coping strategies in the Chilean population. Psicologia: Reflexão e Crítica. 2018;31(1):22.

6.	Babakhani M, Aghabarary M, Norouzinia R. Perceived stress and coping strategies after unsuccessful cardiopulmonary resuscitation among pre-hospital emergency technicians: A multicenter cross-sectional study. Heliyon. 2024;10(10):e31418.
